# Comparing Ugandan motorcycle taxi driver estimations of injury incidence to District-level injury surveillance data as a proxy to determine factors influencing risk perception

**DOI:** 10.11604/pamj.2022.41.177.29363

**Published:** 2022-03-04

**Authors:** Peter Gavin Delaney, Zachary Joseph Eisner, Richard Bamuleke

**Affiliations:** 1 University of Michigan Medical School, Ann Arbor, Michigan, United States of America,; 2 Uganda Red Cross Society, Iganga, Uganda

**Keywords:** Injury, occupational health, risk perception, road trauma, Urban, LMICs

## Abstract

**Introduction:**

road traffic incidents (RTIs) are a leading cause of death among young people, disproportionately affecting low- and middle-income countries (LMICs), where motorcycle taxis disproportionately contribute to injury. Though driver behavior has been identified as the most important factor in RTIs, the factors that influence risk perception, which affect driver behavior, have not been well-studied in LMICs and may inform future strategies to limit risky behavior.

**Methods:**

Ugandan motorcycle taxi drivers (n=117) were surveyed on personal characteristics and experiences, ranking apparent risk of select injury conditions. Rankings were then compared against the actual frequency of corresponding District-level injury surveillance data for the same injury conditions to investigate the accuracy of respondent risk perception. Personal characteristics were then regressed against the perceived risk of certain injury classification rankings to investigate possible factors influencing rankings.

**Results:**

over 26 months, 21,253 injury-related events were recorded in Iganga District, of which 7,424 patient encounters (34.93%) were related to RTI. Ugandan motorcycle taxi drivers tended to over-estimate the risk associated with their profession, but correctly classified the three most common injuries. Regression analyses revealed personal characteristics including personal exposure to RTIs (B=0.037, t=2.035, p=0.044) and years of experience (B=0.026, t=1.828, p=0.070) predicted perceived risk.

**Conclusion:**

Ugandan motorcycle taxi drivers accurately predict the risks associated with their profession. The perception of these risks may be affected by years of experience and previously witnessed RTIs. Further empirical investigation is required to document all key motives and perspectives of drivers as factors that influence risk perception and subsequent behavior in LMICs and may inform future strategies to limit risky behavior.

## Introduction

Road Traffic Incidents (RTIs) are the leading cause of death among people aged 15-29 years, non-fatally injuring between 20 and 50 million people annually [1]. In fact, 90% of the world´s disability-adjusted life years (DALYs) due to RTIs occur in low- and middle-income countries (LMICs), though these countries have fewer registered motorized vehicles than high-income countries [2,3]. Sub-Saharan Africa has the highest RTI fatality rate at 16.6 RTI deaths per 100,000 per year, as compared to the Americas and Europe at 12.4 and 10.3 RTI deaths per 100,000 people per year, respectively [3,4]. With increased motorization, rates of morbidity and mortality will continue to rise, impairing future economic development in LMICs [5,6]. In fact, RTIs are estimated to cost LMICs between 1-2% of their gross national product, estimated at over US$100 billion per year [7].

The proliferation of motorcycle taxi transport as a rapid and reliable service over short- and intermediate distances [5,8] has been attributed to the lack of affordable public transport options in urban and rural African settings, representing a bottom-up response to an absence of government-funded alternatives [9]. This proliferation has also been tied to a subsequent rise in RTIs [10,11]. Among the most vulnerable road users, motorcycle taxi drivers are disproportionately involved in RTIs and are involved in 41% of all RTIs in Uganda [12], while as many as 49.3% and 38.7% of motorcycle taxi drivers report involvement in a road traffic incident during their career while 77.0% and 56.8% report an RTI during their career in surveys in Tanzania and Rwanda, respectively [13,14].

Driver behavior has been identified as the most important factor in RTIs, making it an important area of study to reduce the RTI incidence [15-17]. For experienced drivers, influencing driver behavior to reduce the incidence of RTIs via risk perception appears to be a viable strategy, as increased risk perception has previously been shown to positively influence driver behavior [18]. This is substantiated by evidence of risk perception explaining RTI incidence in cyclists [19], as well as in studies from Cameroon and Denmark in which a lower perception of risk in road users led to negative behavioral outcomes [20,21]. There is a deficit of studies identifying factors that influence risk perception, let alone those influencing motorcycle taxi drivers in LMICs or sub-Saharan Africa [22], essential for identifying factors that influence risk perception and factors that could be amenable to change [18]. In this study, we sought to determine if motorcycle taxi drivers accurately predict the risk associated with their profession and investigate the factors influencing risk perception in Ugandan motorcycle taxi drivers to inform future strategies intended to limit risky driver behavior.

## Methods

**Aim:** as driver behavior has been identified as the most important factor in RTIs, understanding factors that influence risk perception, which affect driver behavior, may inform future strategies to limit risky behavior. To explore the factors that contribute to perception of risk in motorcycle taxi drivers, Ugandan motorcycle taxi drivers were surveyed on personal characteristics and experiences and asked to rank the perceived risk of certain injury classifications. To understand if participants accurately perceived the relative risk of seven injury classifications, their responses were then compared to the actual incidence of the corresponding injury classifications based on data collected from the District Health Office (DHO). Results were then regressed using simple and multiple linear regressions against the perceived risk of certain injury classification rankings to investigate possible factors influencing their rankings, which functioned as a proxy for risk perception.

**Setting:** according to the 2010 Global Burden of Disease study, Uganda ranks fifth of 15 sub-Saharan African countries with the highest DALYs due to RTIs [1,23]. RTIs are the leading cause of death in Uganda, with a fatality rate of 28.9 RTI deaths per 100,000 people [5].

**District-level health facility data acquisition and analysis:** the Ugandan Ministry of Health´s (MOH) “Health Management Information System Form 105/108: Health Unit Outpatient Monthly Report” was accessed and collected from the District Health Office´s (DHO) health records for Iganga District for analysis. The form reports seven injury classification categories tracked by the Ugandan MOH, including (1) “Injuries - Road Traffic Accidents” and (2) “Injuries due to motorcycle (boda-boda),” and non-RTI-related injury classifications including (3) “Jaw Injuries,” (4) “Injuries due to Gender based violence,” (5) “Injuries (Trauma due to other causes),” (6) “Animal bites,” and (7) “Snake Bites.” We transferred anonymous population-level data from monthly forms for each of the 58 registered health facilities in Iganga District, spanning from January 2014 to February 2016, into an electronic database for analysis. Based on the data, the seven injury classifications were ranked by total frequency over the 26-month period for comparison against the respondents´ perceived rankings of the seven classifications´ perceived frequency.

**Representative sample determination and study participant selection:** in accordance with a standard convenience sampling method previously developed for use with motorcycle taxis in Uganda, we generated a sample of motorcycle taxis determined to be adequately representative of motorcycle taxis in the municipality of Iganga [24]. A municipal motorcycle taxi driver registry revealed a population of N=2,178 motorcycle taxis operating in the municipality. The sample size formula for estimating p with a bound on the error of estimation of magnitude B was utilized (Equation 1).

Equation 1: Sample size required to estimate p with a bound B on the error of estimation:


n=Npq/[(N−1)D+pq]

Where q = 1 - p and D = (B^2^)/4

To estimate conservative sample size, p was set = 0.5 and the bound on the error of estimation was set at 10% to control for motorcycle taxi movement and operation inside and out of the municipality, as well as errors in record-keeping. Thus, a sample size of at least n=96 motorcycle taxi drivers (95.65) was determined to be adequate for reliable results and sufficiently representative of motorcycle taxis in the municipality. Participants were randomly selected from the municipal association´s driver registry listing and contacted. A total of 117 of 125 (94%) motorcycle taxi drivers contacted via telephone consented to the survey, at which point they were instructed to meet for survey administration.

**Survey instrument:** the survey instrument was composed of eight items: (1) driver´s stage/staging location; (2) age; (3) years of experience as a motorcycle taxi driver; (4) age at which the respondent began working as a motorcycle taxi; (5) education level; (6) number of road traffic injuries witnessed over the past six months; and (7) deaths witnessed over the past six months due to road trauma. The final item of the survey (8) had participants rank the seven injury classification categories tracked by Ugandan MOH by perceived frequency of incidence, and thus determine which injury classification they perceived as most risky, for analysis and comparison to the actual frequencies obtained from the Ugandan DHO´s data. The survey instrument was administered on-site with participants recording responses on paper. For illiterate drivers, data collectors read the questions aloud and marked verbal responses. Motorcycle taxi drivers arrived at their convenience over the course of a single day to complete the survey.

**Data analysis:** to compare motorcycle taxi driver risk perception to the actual ranking based on the frequency of corresponding injury classifications from data obtained from the Ugandan DHO, the median risk ranking by participants and interquartile range (IQR) were calculated from the survey for each injury classification category in R (R Foundation for Statistical Computing, Vienna, Austria). Simple and multiple linear regression were used to construct a model for motorcycle taxi driver risk perception from the six personal characteristics and experiences collected in surveys, namely: (1) age; (2) years of experience; (3) starting age; (4) education level; (5) RTI injuries witnessed in the past six months; (6) deaths witnessed in the past six months.

For the risk perception scores of each injury classification, respondents assigned a ranked value between 1 and 7 for comparison to the true ranked list of the seven injury classifications based on the data collected from the District Health Office and generated from the total frequencies of injuries over the 26-month period investigated. A lower value assigned to an injury classification by a respondent indicated they felt it to be more prevalent (1^st^ versus 7^th^). Values assigned by respondents were totaled and averaged to obtain rankings of the aggregate perceived risk concerning injury classifications of the sample and then compared to the actual ranking based on the frequency of corresponding injury classifications to determine the difference between the perceived rank of injuries to the real rank of injury classifications from Ugandan government data. For regression analysis, rankings were reversed (1^st^ became 7, 7^th^ became 1) so the proper directionality would be maintained. To further measure association between variables, Pearson´s correlation coefficient was determined between risk perception and personal characteristics.

**Ethical clearance:** ethical approval for the study was granted by TASO and the Uganda National Council on Science and Technology (Kampala, Uganda). Permission to conduct the study was also sought and obtained from the municipal and district leadership of the boda-boda driver associations, as well as the Local Consulate V (LCV) and the Mayor of Iganga.

## Results

### District-level descriptive injury statistics

**Figure 1:**
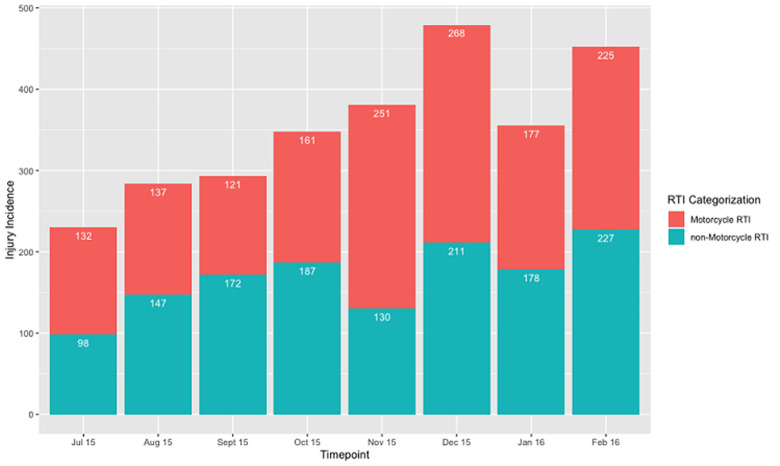
total cases of injuries due to RTIs distinguishing motorcycle taxi-related injuries

There were 21,253 total injury-related events recorded between January 2014 and February 2016 in Iganga District, of which 7,424 patient encounters (34.93%) were injuries related to road traffic injuries, second only to non-defined “other” injuries (Table 1). While injuries resulting from road traffic incidents represent 34.93% of all injuries in Iganga District alone (with over half involving motorcycle taxis), the two largest categories, injuries due to road traffic incidents and injuries due to “other causes,” together comprise 90.75% of the injury encountered in Iganga District. Of reported injuries, beginning in July 2015, monthly reports distinguish between general road traffic incidents and those involving motorcycle taxis, given their prevalence across Uganda. Figure 1 represents the split between the proportion of RTIs not involving motorcycle taxis and those did. Over that period, motorcycle taxi-related injuries represented the majority (52.50%) of all RTI injuries in Iganga.

**Table 1:** breakdown of injuries sustained in Iganga over a 26-month period

	By gender		By age		By gender and age				Total
					Male	Male	Female	Female	
	Male	Female	0-4	5+	0-4	5+	0-4	5+	
Jaw injuries	205	204	10	399	4	201	6	198	409 (1.9%)
Injuries- road traffic incidents	4,304	3,120	970	6,454	459	3,845	511	2,609	7,424(34.9%)
Injuries due to gender-based violence	354	499	38	815	15	339	23	476	853 (4.0%)
Injuries (trauma due to other causes)	5705	6159	2084	9780	992	4713	1092	5067	11,864 (55.8%)
Animal bites									
Domestic	165	155	36	284	13	152	23	132	320 (1.5%)
Wild	8	4	1	11	0	8	1	3	12 (0.1%)
Insects	27	55	10	72	5	22	5	50	82 (0.4%)
Snake bites	130	159	28	261	16	114	12	147	289 (1.4%)
**Total**	10898	10355	3177	18076	1504	9394	1673	8682	**21,253**

### Study participant survey demographics

**Figure 2:**
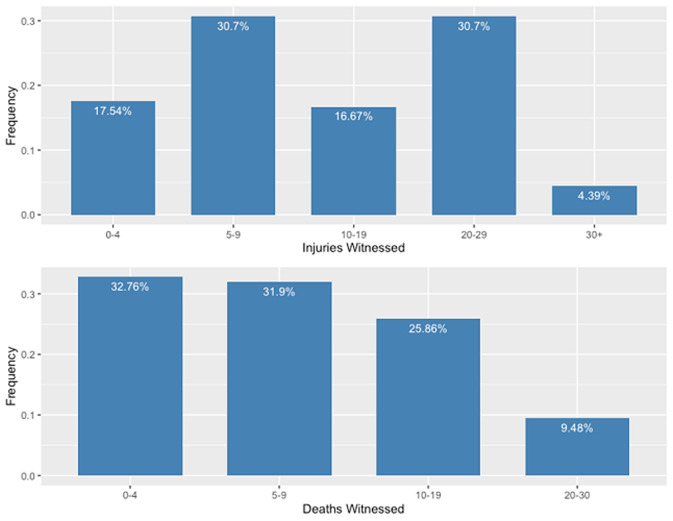
number of injuries and deaths witnessed in the past six months by study participants

The 117 motorcycle taxi drivers who completed the survey represented 46 of the 53 stages/staging locations in the municipality (Figure 2). All motorcycle taxi drivers were male, ranging in age from 18 to 67 years with a median age of 32 years. Experience as a motorcycle taxi driver ranged from 1 to 27 years with a median of 7 years, while the median age at which the study participants began as motorcycle taxi drivers was 26 years, ranging from 15 years to 56 years. Over half of study participants had completed some form of secondary education (Table 2). Over the six months prior to the study, 17.5% (n=20) of study participants reported having witnessed 0 and 4 RTIs, 30.7% (n=35) of study participants reported having witnessed between 5 and 9 RTIs, 16.7% (n=19) of study participants reported having witnessed between 10 and 19 RTIs, 30.7% (n=35) of study participants reported having witnessed between 20 and 29 RTIs, and 4.4% (n=5) of study participants reported having witnessed more than 30 RTIs. Additionally, 32.8% (n=38) of study participants reported having witnessed between 0 and 4 deaths, 31.9% (n=37) of study participants reported having witnessed between 5 and 9 deaths, 25.9% (n=30) of study participants reported having witnessed between 10 and 19 deaths, and 9.5% (n=1) of study participants reported having witnessed between 20 and 30 deaths (Figure 2). Frequencies derived from the DHO data aggregated from monthly reports show the ranked order of injury prevalence to be: “Other Injuries,” “Motorcycle Taxi Injuries,” “General Road Traffic Injuries,” “Gender-Based Violence Injuries,” “Animal Bites,” “Jaw Injuries,” “Snake Bites.” Motorcycle taxi driver study participants, however ranked “Motorcycle Taxi Injuries” as being most prevalent, followed by: “General Road Traffic Injuries,” “Gender-Based Violence Injuries,” “Jaw Injuries,” “Other Injuries,” “Animal Bites,” and “Snake Bites.”

**Table 2:** demographic characteristics of survey respondents

Characteristic	Response (n)	Response (%)
**Gender**		
Male	117	100.00%
Female	0	0
**Age**		
18 to 19 years	2	1.71%
20 to 24 years	6	5.13%
25 to 29 years	33	28.21%
30 to 34 years	29	24.79%
35 to 39 years	26	22.22%
40 to 44 years	9	7.69%
45 to 49 years	8	6.84%
50 to 54 years	2	1.71%
Over 55 years	2	1.71%
**Education**		
None	3	2.73%
Primary	43	39.09%
Secondary	61	55.45%
University	3	2.73%
**Years of Experience**		
1 to 4 years	37	32.17%
5 to 9 years	44	38.26%
10 to 14 years	24	20.87%
15 to 19 years	5	4.35%
20 to 24 years	4	3.48%
Over 25 years	1	0.87%
**Starting Age**		
15 to 19 years	16	14.04%
20 to 24 years	40	35.09%
25 to 29 years	26	22.81%
30 to 34 years	23	20.18%
35 to 39 years	6	5.26%
40 to 44 years	1	0.88%
45 to 49 years	1	0.88%
Over 50 years	1	0.88%

### Statistical analyses

The results of the linear regression demonstrated statistically significant results for two of six study participant characteristics after they were regressed against the injury classification category rankings for the perceived risk of motorcycle taxi transport. The linear regression analysis revealed “number of injuries witnessed” and “years of experience as a motorcycle taxi” significantly predicted motorcycle injury risk perception. As participants witnessed more RTIs, they perceived increasing risk related to motorcycle injuries (B = 0.037, t = 2.035, p = 0.044). Additionally, as participants accrue experience as a motorcycle taxi driver, the perceived risk of motorcycle injuries increases (B = 0.026, t = 1.828, p = 0.070). Multiple linear regression showed that years of experience (1) and amount of RTIs witnessed (2) may also be used in tandem to model trends in perceptions of motorcycle taxi risk (B1 = 0.035, B2 = 0.022, t1 = 1.27, t2 =1.54, p = 0.053). Deaths due to road traffic incidents witnessed, a motorcycle taxi driver´s starting age, level of education, and the age of the participant did not significantly predict motorcycle injury risk perception. Pearson´s correlation coefficient was determined for the association between the variables “injury classification category rankings for the perceived risk of motorcycle injuries” and “number of injuries witnessed” or “years of experience.” The number of injuries witnessed by participants was significantly correlated with perceived risk (r = 0.187, p = 0.04) and a strong trend was noted between years of experience as a motorcycle taxi driver and perceived risk (r = 0.170, p = 0.07).

## Discussion

Findings suggest that personal experience influences risk perception in motorcycle taxis in Uganda, a low-income, sub-Saharan African country, similar to studies of road users in high-income countries [18]. In Ugandan motorcycle taxi drivers, the number of injuries witnessed and years of experience as a motorcycle taxi driver appear to significantly influence risk perception. Study participants tended to overestimate the risk of motorcycle taxi injuries in their rankings of injury classification category prevalence, as median respondent rankings for the prevalence of motorcycle RTIs (1^st^) were slightly higher than the actual motorcycle injury category ranking (2^nd^) by total frequency informed by district-wide injury data. This may be due to the nature of the profession of study participants, such that working as a motorcycle taxi driver would provide added exposure to RTIs, leading to an undue prioritization of the category. Our findings lend credence to this notion, as motorcycle taxi-related injuries represented the majority (52.50%) of all RTI injuries in Iganga over the 26-month period studied, similar in prevalence to other African settings [25]. Alternatively, the overestimation of motorcycle taxi-driven RTIs could be due in part to participants acknowledging the social stigmatization surrounding their profession, as motorcycle taxis are often seen as reckless, informal alternatives to non-existent public transportation in Uganda [26,27]. The willingness of study participants to acknowledge the danger associated with their profession [28] and then still operate in the same stigmatized profession merits further investigation. Overall, participants overestimated general RTIs compared to the injury classification category “other injuries”, most likely due to not initially considering the many ways one may encounter injury through “other” means. This aside, study participants successfully perceived and sequentially ranked the three top injury classification categories (motorcycle taxi-related injury, general road traffic injuries, and gender-based violence-related injuries), indicating participants possess an accurate perception of relative injury classification risk.

The objective of elucidating the factors that contribute to risk perception, given the demonstrated impact of risk perception on resultant driving behavior, is to help promote safer driving practices by informing the content that may be used to encourage safer driving behavior, reduce RTI incidence, and subsequently make transportation safer in LMICs. Previous investigators have advocated for the development of educational tools that might be useful for promoting not only the avoidance of risky behaviors, but also a generalized awareness on road safety issues, as knowledge of traffic norms was shown to be a contributory factor to RTIs [19]. Evidence supports the hypothesis of a mediation between risk perception and risky behaviors to improve traffic safety, consistent with RTIs emerging as a global public health issue that must be tackled by a multidisciplinary approach [29-31]. Identifying factors that influence risk perception and the factors that could be amenable to change to improve road safety initiatives and provide information and support to counter positive factors has been advocated for by previous investigators [18]. Our findings revealed risk perception may be influenced by years of experience in the profession, supporting previous findings that demonstrate the importance of experience on lowering crash risk [32].

In a recent intervention, investigators in Nepal sought to harness self-determination theory and the Health Belief Model to test the impact of short video messages on influencing perceived susceptibility, severity, and driving behavior. In doing so, they demonstrated that directive messages influenced an individual to value the recommended behavior, which in turn influenced perceived susceptibility, severity, and behavior [33]. Given that our findings suggest exposure to an increasing number of injuries leads to increased risk perception, examining if similar visual media forms may also influence risk perception in road users like motorcycle taxi drivers in LMICs merits further study.

Contrary to prior studies that demonstrate age influences motorcyclists´ attitudes and behaviors in traffic or exerted an important effect in the variation of the explanatory structure of associated models, the age of participants in this study was not found to be a significant factor influencing risk perception [19,34-36]. These studies examined different samples (cyclists vs motorcyclists) from different geographic locations (Latin American, European, and North American vs Uganda) with different income levels (upper/middle-income countries vs low-income country), however. It is possible that such demographic variations explain the inconsistency of age as a factor contributing to risk perception in our study.

Investigators attempted to limit bias through a random selection of drivers using a random number generator from the municipal association´s driver registry listing, however, there is a chance the sampling was not representative of the municipal motorcycle taxi driver population. Although sampling was random, systematic, and in sufficient quantity to be representative, some of the trends noted may have become more significant with further powering the study with a larger sample. Expanding the characteristics and personal experiences surveyed would be helpful in future studies to explore the influence of other factors as well, as investigators felt those currently included were most apropos and relevant to the study in question. Specifically, including personal RTI history in the survey would have been particularly useful in comparing findings to those of Ngueutsa and Kouabenan (2017), who counter intuitively found participants reporting involvement in a severe accident perceived road travel as less risky compared with those involved in fewer, or less severe accidents [20]. This seems dichotomous compared to the results of this study, suggesting the number of RTIs witnessed influences risk perception, as personal accident history and number of road traffic incidents witnessed appear to influence risk perception in opposite directions, an area meriting further study.

## Conclusion

Given the demonstrated impact of risk perception on resultant driving behavior, elucidating the factors that contribute to risk perception is essential for promoting safer driving practices that reduce RTIs. In Ugandan motorcycle taxi drivers, the number of injuries witnessed and years of experience as a motorcycle taxi driver appear to influence risk perception. Ugandan motorcycle taxi drivers also accurately predict the risk of injury associated with their profession. Further empirical investigation is required to document all the key motives and perspectives of drivers and their behaviors in low- and middle-income countries when endorsing policy change and program development.

### What is known about this topic

- *Driver behavior has been identified as the most important factor in RTIs;*
- *Increased risk perception has previously been shown to positively influence driver behavior;*
- *Though driver behavior has been identified as the most important factor in RTIs, the factors influencing risk perception, which affect driver behavior, have not been well-studied in LMICs and may inform future strategies to limit risky behavior*.

### What this study adds

- *In this study, we sought to determine if motorcycle taxi drivers accurately predict the risk associated with their profession and investigate the factors influencing risk perception in Ugandan motorcycle taxi drivers to inform future strategies intended to limit risky driver behavior;*
- *Ugandan motorcycle taxi drivers accurately predict the risk of injury associated with their profession;*
- *In Ugandan motorcycle taxi drivers, the number of injuries witnessed and years of experience as a motorcycle taxi driver appear to influence risk perception*.
